# Uncertainty quantification in multi-class image classification using chest X-ray images of COVID-19 and pneumonia

**DOI:** 10.3389/frai.2024.1410841

**Published:** 2024-09-18

**Authors:** Albert Whata, Katlego Dibeco, Kudakwashe Madzima, Ibidun Obagbuwa

**Affiliations:** ^1^Department of Mathematical Sciences, Sol Plaatje University, Kimberley, South Africa; ^2^Department of Computer Science and Information Technology, Sol Plaatje University, Kimberley, South Africa

**Keywords:** uncertainty quantification deep neural networks, Bayesian neural networks, Monte Carlo dropout, Ensemble Monte Carlo, chest-X-ray, classification metrics, multi-class classification

## Abstract

This paper investigates uncertainty quantification (UQ) techniques in multi-class classification of chest X-ray images (COVID-19, Pneumonia, and Normal). We evaluate Bayesian Neural Networks (BNN) and the Deep Neural Network with UQ (DNN with UQ) techniques, including Monte Carlo dropout, Ensemble Bayesian Neural Network (EBNN), Ensemble Monte Carlo (EMC) dropout, across different evaluation metrics. Our analysis reveals that DNN with UQ, especially EBNN and EMC dropout, consistently outperform BNNs. For example, in Class 0 vs. All, EBNN achieved a *U*Acc of 92.6%, *U*AUC-ROC of 95.0%, and a Brier Score of 0.157, significantly surpassing BNN's performance. Similarly, EMC Dropout excelled in Class 1 vs. All with a *U*Acc of 83.5%, *U*AUC-ROC of 95.8%, and a Brier Score of 0.165. These advanced models demonstrated higher accuracy, better discriaminative capability, and more accurate probabilistic predictions. Our findings highlight the efficacy of DNN with UQ in enhancing model reliability and interpretability, making them highly suitable for critical healthcare applications like chest X-ray imageQ6 classification.

## 1 Introduction

Computer vision has made enormous progress in recent times. The development of advanced deep learning techniques for computer vision is motivated by the human visual system, which is one of the richest senses that we have. While computer vision aims to replicate the capabilities of the human visual system, it is important to acknowledge that achieving this goal is still a considerable distance away. Thus, deep learning algorithms that can achieve state-of-the-art performance are still required for computer vision problems such as face recognition (Schroff et al., 2015), object detection (Ren et al., 2015), and image classification (Krizhevsky et al., 2012). These algorithms are well suited to analyze images and signals. Machine learning (ML) refers to a collection of expert systems that encompass the creation of expert computer systems capable of learning from their mistakes and improving their performance, as described by Novaković et al. (2017). Traditional machine learning algorithms, in contrast to deep learning algorithms, which may learn representations directly from raw data, rely on examples expressly constructed by human specialists to represent specific problem areas. Applying machine learning (ML) techniques to computer vision and image analysis systems encounters several challenges, including handling noisy and imperfect images, addressing complex background removal, and accommodating variations in illumination. These factors pose difficulties in developing handcrafted representations suitable for supervised machine learning algorithms.

Deep learning (DL) algorithms provide a viable solution to the difficulties faced by traditional machine learning (ML) models that manually extract features from images. The inherent multi-layered processing architecture of deep learning algorithms empowers these algorithms to autonomously learn and derive representations, thereby circumventing the limitations associated with manual feature extraction.

This study focuses on utilizing DL to distinguish between chest X-ray images associated with pneumonia, COVID-19, and normal cases. In order to classify these chest X-ray images and return the probability of an instance falling into a particular class, a considerable number of training examples that comprise healthy images (with no pneumonia nor COVID-19) or infected (with pneumonia or COVID-19) are utilized.

Deep learning-based techniques have demonstrated remarkable performance in distinguishing chest X-ray images that are infected with pneumonia or COVID-19 and those that are normal. The development of deep learning-based techniques for disease detection in real-life scenarios may face challenges due to the potential drawback of overconfident diagnostics, despite achieving high classification results (Hernández and López, 2020). The reason behind the overconfidence is that most state-of-the-art deep learning models often fail to provide information about uncertainties, such as epistemic uncertainty (uncertainty stemming from the model itself) and/or aleatoric uncertainty (uncertainty arising from the data). Therefore, important features and sensitive information can be lost if traditional machine learning outcomes that do not account for either aleatoric or epistemic uncertainty are trusted (Abdar et al., 2021b). Furthermore, deep learning methods are not designed to account for the uncertainty in a model's predictions, nor are they able to identify the important features that are responsible for a specific prediction (Abdar et al., 2021b). The “inner workings” of these deep learning methods are not understood as they are typically used as “black boxes.”

This study seeks to gain better insights about deep learning models by quantifying the uncertainty that is inherent in these models when they are applied in computer vision tasks. We employ the Bayesian Deep Learning (BDL) techniques as well as the Deep Neural Network (DNN) that is coupled with the widely used method, the Monte Carlo Dropout (MCD), and other dropout techniques to quantify uncertainty. The MCD approach is better than the computationally expensive techniques that include the Markov Chain Monte Carlo (MCMC) (Kendall and Gal, 2017). Moreover, the use of Bayesian uncertainty quantification (UQ) techniques such as MCD produces well-calibrated and precise estimates of uncertainty (Hernández and López, 2020). It is important to note that the Bayesian UQ techniques use a distinct approach that derives the posterior probabilities of the parameters (weights) as opposed to traditional deep learning estimates that generate point estimates.

This study presents an innovative approach to uncertainty quantification in deep learning models used in medical image analysis. By focusing on multi-class classification of chest X-ray images, it aims to improve the interpretability and reliability of these models. Therefore, to advance UQ using Bayesian methods this study specifically makes the following significant contributions:

(i) Adapt the innovative concept of a binary uncertainty confusion matrix, along with its novel performance metrics proposed by Asgharnezhad et al. (2022), for objective uncertainty quantification. This adaptation extends the binary uncertainty confusion matrix with its novel performance metrics for multi-class tasks, enhancing the evaluation of model performance and reliability across various classes.
(ii) Provide valuable insights into the performance and reliability of different uncertainty quantification models across various classes.
(iii) Provides a comprehensive evaluation of uncertainty quantification techniques that include Bayesian neural networks (BNN), Monte Carlo dropout, Ensemble Bayesian Neural Network (EBNN), Ensemble dropout, and Ensemble Monte-Carlo (EMC) dropout. The aim is to compare the effectiveness of these methods in capturing and quantifying uncertainty in the predictions of multi-class classification models for chest X-ray images. This will give valuable insights into the performance and reliability of different uncertainty quantification techniques across various classes. Such information would assist practitioners when they are selecting appropriate uncertainty quantification methods for their neural network models.
(iv) Highlights the potential of uncertainty-aware models to enhance the reliability and interpretability of predictions in critical medical image analysis. This will in turn help to improve the safety and efficacy of AI-driven healthcare solutions, particularly in the classification of COVID-19 cases.

## 2 Related works

We note that machine learning methods have achieved great success in solving many real-life problems, but they have not been able to provide more information about the reliability of their predictions in most cases. This challenge has necessitated the use of the promising Bayesian neural networks (BNNs) which model prior distributions on the model parameters to quantify uncertainty (Alarab and Prakoonwit, 2023). The authors indicate that assigning a prior distribution over a model's parameters and then marginalizing the parameters creates a predictive distribution that uses Bayesian averaging. With this framework, prior distributions are assigned to the weights of the model, and thereafter, Bayes' theorem is used to determine the posterior distributions which are approximated because they cannot be evaluated analytically. Bayesian UQ techniques have been used in several image classification tasks (Harakeh et al., 2020; Kwon et al., 2020; Bessai-Mechmache et al., 2022). However, Monte Carlo (MC) sampling has emerged as an effective technique that can be used to estimate the posterior distribution and thereby quantify uncertainty (Neal, 2012). The use of MC sampling has a limitation in that when it is deployed in deep architectures, it can be slow and computationally expensive. Gal and Ghahramani (2016) state that this limitation can be addressed by employing efficient techniques such as MC-dropout, which has been developed as a regularization technique to quantify uncertainty and avoid overfitting. Furthermore, the authors highlight that applying the dropout regularization technique after each hidden layer allows the MC-dropout technique to evaluate uncertainty in neural networks. Moreover, the MC-dropout technique is employed during the testing phase to generate uncertainty-aware estimates (Alarab and Prakoonwit, 2023). Alarab and Prakoonwit (2023) demonstrated that MC-dropout was more effective in quantifying uncertainty when applied to the Elliptic (Bitcoin-derived) dataset compared to other techniques. Lemay et al. (2022) indicated that the predictions derived from the Monte Carlo dropout were better calibrated when it was employed on four medical image classification tasks that used DenseNet and ResNet architectures. According to the authors, the output probabilities produced were more accurate and reflected the likelihood of correct classification. Mobiny et al. (2021) proposed the Monte Carlo DropConnect (MC-DropConnect) technique that incorporated Bayesian Inference in deep neural networks (DNNs). In this approach, the weights/parameters were assumed to follow a Bernoulli distribution. The empirical results showed that the predictive accuracy of MC-DropConnect significantly outperformed other state-of-the-art techniques. The multi-class classification based Monte Carlo-based adversarial attack (MC-AA) method on the Cora dataset was introduced by Alarab and Prakoonwit (2023). The authors compared MC-AA with other recent uncertainty models such as, convolutional neural networks (CNN) and LeConv. The best results for modeling uncertainty were obtained using LeConv (AUC = 0.889) deployed on the Cora datasets and CNN (AUC = 0.98) deployed on the MNIST datasets.

Thiagarajan et al. (2021) classified histopathological images using a hybrid Bayesian-convolutional neural network (Bayesian-CNN). When applied to a large portion of the test dataset, the Bayesian-CNN used the quantified uncertainties to significantly enhance the performance of the CNN. Abdar et al. (2021b) employed techniques such as EMC dropout and deep ensemble for uncertainty quantification in skin cancer image classification. Abdar et al. (2023) employed the effective Ensemble MC Dropout (EMCD) technique, achieving a prediction accuracy of 99.08% for the computed tomography (CT) scan dataset and 96.35% for the chest X-ray dataset. The authors also indicate that EMCD was used not only to detect COVID-19 but also to quantify uncertainty using chest X-ray images. McDermott and Wikle (2019) employed deep ensemble (DE) to quantify uncertainty. DE uses an ensemble model that comprises several neural networks. The authors used a DE echo state network model for spatio-temporal forecasting while also evaluating and quantifying uncertainty. DE methods have been found to outperform the Bayesian neural networks in uncertainty quantification, yielding more accurate UQ estimates (Alarab and Prakoonwit, 2023). However, Abdar et al. (2021b) noted that DE methods tend to be more computationally expensive.

Narlı (2021) investigated the impact of applying local histogram equalization (LHE) on the performance of deep learning architectures for COVID-19 classification using chest X-ray images. The effect of the disk factor in LHE on transfer learning was examined by comparing the results obtained with and without LHE preprocessing. The dataset used by Narlı (2021) consisted of chest X-ray images from three classes: COVID-19, Pneumonia, and Normal. Each chest X-ray image was segmented into two parts: the right lung lobe and the left lung lobe. The classification performance of transfer learning was evaluated by applying different disk values for LHE and the experiments were conducted using various pre-trained DL architectures, including VGG16, AlexNet, and Inception models. Altan and Narlı (2022) employed simplistic CNN architectures with enhanced medical images using contrast limited adaptive histogram equalization (CLAHE) to classify of healthy chest X-rays (CXRs) and those with COVID-19. The study utilized a large-scale dataset of 3,615 COVID-19 cases, demonstrating the clinical applicability of the proposed method, which enhanced feature learning and preprocessing stages to facilitate early diagnosis of COVID-19. The study achieved an impressive accuracy rate of 95.878% for binary classification of COVID-19 and healthy cases using the VGG16 model with optimal CLAHE parameters.

Yang and Fevens (2021) conducted experiments using two medical imaging datasets: a SARS-CoV2 CT dataset and the BreaKHis dataset (Spanhol et al., 2015). The study highlighted the ability to identify uncertain samples and categories, demonstrating that by excluding a percentage of the most uncertain inputs, the accuracy of the model's predictions could be significantly improved. This approach ensured better clinical outcomes by providing a more reliable framework for the application of DNNs in medical diagnostics. Moreover, the findings underscored the potential of UQ methodologies to enhance the practical utility of DNNs in healthcare, ultimately supporting better patient management and treatment strategies.

Machine learning models have typically been evaluated in biomedical research using measures such as sensitivity, specificity, precision, accuracy, and Matthews correlation coefficient (MCC). Rabiei et al. (2022) used sensitivity, specificity, and accuracy metrics to evaluate machine learning models for predicting breast cancer recurrence, demonstrating their effectiveness in accurately identifying true positive and true negative cases, which is crucial for clinical decision-making. Similarly, Helaly et al. (2022) used deep learning models to detect early Alzheimer's disease and measured sensitivity, specificity, precision, and AUC-ROC. The inclusion of AUC-ROC enabled a detailed assessment of the models' discriminative performance across various threshold settings, offering a robust evaluation framework. Hajian-Tilaki (2013) discussed the application of sensitivity, specificity, precision, and accuracy within the context of ROC curve analysis for medical diagnostic test evaluation, highlighting their importance in capturing trade-offs between true positive and false positive rates and offering a comprehensive tool for evaluating diagnostic accuracy. However, traditional evaluation metrics do not account for uncertainty in models. It is important to derive and use performance metrics that quantify uncertainty, as understanding model uncertainty can significantly enhance the reliability and interpretability of predictions in real-world applications. Calculating uncertainty-aware evaluation metrics is crucial as it can boost confidence and trust in machine learning models. Several deep learning models struggle to provide necessary uncertainty-aware predictions, as they often fail to capture inherent uncertainties effectively. Consequently, these models lack the required uncertainty-aware reasoning when deployed in computer vision tasks. To address this limitation, it is essential to explore methodologies that effectively quantify uncertainty within deep learning models. Therefore, this study investigates the application of Bayesian methods to determine if they offer improved uncertainty quantification in deep learning techniques for multi-class classification of chest X-ray images. Uncertainty quantification (UQ) in multi-class classification has not received much attention, as UQ research primarily focuses on regression and binary classification tasks, overlooking the unique techniques and challenges specific to multi-class classification.

The remainder of the paper is organized as follows. Related works are presented in Section 2. The proposed methodology is described in Section 3.1. The experiments are introduced in Section 3.8. Section 4 discusses the results and Section 6 concludes the paper.

## 3 Materials and methods

### 3.1 Dataset

This paper aimed to achieve significant advancements in uncertainty quantification through multi-class classification using a publicly available chest X-ray image dataset, accessible at https://www.kaggle.com/datasets/prashant268/chest-xray-covid19-pneumonia. The dataset comprised 619 chest X-ray images of patients with COVID-19, 526 chest X-ray images of patients with pneumonia, along with 732 images depicting healthy/normal lungs. We noted that there was a moderate imbalance in the dataset and we applied the resampling technique to achieve balance by oversampling the minority class. For this, we used the *imblearn* library in Python. Specifically, the *RandomOverSampler* was employed to oversample the minority classes to obtain the following class distributions: Class 1 (COVID-19 images) 732, Class 0 (Normal images) 732, and Class 2 (Pneumonia images) 732.

### 3.2 Multi-classification

In real-life scenarios, numerous classification problems involve the need to distinguish between more than two classes. Examples of such problems include face recognition, hand gesture recognition, general object detection, speech recognition, and many others. These applications require algorithms and techniques that can effectively classify data into multiple distinct categories, enabling various tasks and applications in fields such as computer vision, natural language processing, and human-computer interaction.

In this paper, we adopt a methodology for addressing multi-class classification tasks referred to as the “One vs. All” (OvA) strategy. Under this approach, we train multiple linear classifiers *C*, where *C*>2. *C* denotes the number of classes within the classification task. Each classifier is tasked with distinguishing one class from the remaining classes in the dataset. Therefore, this method entails the training of *C* binary classifiers, each dedicated to distinguishing a single class from the rest. During training, for each class, a binary target variable is created. We assign a positive label to instances belonging to the class being considered and a negative label to instances belonging to other classes. Then, we train a linear classifier using this binary target variable and the corresponding features. At prediction time, we apply all *C* classifiers to the input data, and each classifier produces a score or probability indicating the likelihood of the input belonging to its respective class. The class with the highest score is assigned as the predicted class for the input. This technique effectively reduces the multi-class problem into a series of binary classification problems. Each classifier learns to discriminate one class from the rest, allowing us to handle problems with more than two classes using linear classifiers.

In a dataset with multiple classes, denoted as ${\left(x_{n},y_{n}\right)}_{N-1}^{N}$, there exist *C* distinct classes of data. Similar to the two-class scenario, we have the flexibility to use any *C* distinct labels for distinguishing between these classes. For convenience's sake the following label values *y*_*n*_ ∈ {0, 1, ..., *C*−1} are assigned.

### 3.3 Deep learning methods

#### 3.3.1 Bayesian neural networks

In this section, we provide a brief overview of Bayesian neural networks (BNNs). BNNs are robust against overfitting and capable of handling high-dimensional inputs, such as images (Abdar et al., 2021b).

A neural network is considered as a probabilistic model when it is able to account for and quantify uncertainty within its predictions. A probabilistic neural network can be represented by *P*(*y* ∣ *x*, ω), where *X* = {*x*_1_, ..., *x*_*n*_} are the training samples (input data), and *Y* = {*y*_1_, ..., *y*_*n*_} represents the set of all possible outcomes (output data). In addition, we consider **ω**, to be the set of parameters that are learnt through a complete Bayesian approach. Furthermore, using a training dataset $D={\left\{X,Y\right\}}_{i=1}^{N}$, the posterior distribution, *P*(ω ∣ *D*) is evaluated through Bayesian inferenece that employs marginalization over all the values of ω (Khairnar et al., 2020). Therefore, to estimate *P*(ω ∣ *D*) the Bayes theorem is employed as follows:


(1) $$\begin{array}{l} P\left(\omega |D\right)=\frac{P\left(D|\omega \right)P\left(\omega \right)}{P\left(D\right)} \end{array}$$

where the likelihood of training the data *D* given the parameters ω is given by *P*(*D* ∣ ω) and, $P\left(D|\omega \right)=\prod_{n=1}^{N}P\left({ŷ}^{n}|{\hat{x}}^{n},\omega \right)$ is the product of the likelihoods, assuming that each training data point is independently and identically distributed (*iid*). (${\hat{x}}^{n},{ŷ}^{n}$) represents, respectively, the input data and associated labels. The term *P*(ω) denotes the distribution of weights before observing the data, and $P\left(D\right)=\int_{{\text{Ω}}_{\omega }}P\left(D|\omega \right)P\left(\omega \right)d\omega$ denotes the marginalization over the weight distribution. Equation 1 shows how well the parameters ω explain the training data that was observed.

Once *P*(ω ∣ *D*) has been determined, the expected values of the predictive distributions can be used to obtain predictions for test data. Thus, for an unknown label ŷ of a data observation $\hat{x}$, the predictive distribution can be expressed as:


(2) $$\begin{array}{l} P\left(ŷ|\hat{x}\right)={𝔼}_{P\left(\omega |D\right)}[P\left(ŷ|\hat{x},\omega \right]=\int_{{\text{Ω}}_{\omega }}P\left(ŷ|\hat{x},\omega \right)P\left(\omega |D\right)d\omega . \end{array}$$

The Bayes approach aims to optimize the parameters, ω, by maximizing the likelihood, *P*(*y* ∣ *x*, ω). In this study, our task involves multi-classification of images, so we utilize the softmax (Liu et al., 2016) likelihood to compute the predictive probabilities as follows:


(3) $$\begin{array}{l} P\left(y=k|x,\omega \right)=\frac{expf_{k}^{\omega }\left(x\right)}{\sum_{j=1}^{K}expf_{j}^{\omega }\left(x\right)} \end{array}$$

For classification purposes, the model's output can be obtained using a softmax function this allows sampling from the probability vector: $P\left(y=k|x,\omega \right)=\text{softmax}\left(f^{{ŵ}_{t}}\left(x\right)\right)$. The resulting model output is then mapped to a set of class labels for multi-classification.

The computation of the posterior predictive probabilities, *P*(*y* = *k* ∣ *x*, ω), as depicted in Equation 4, presents a significant challenge as it cannot be evaluated analytically. This is because it requires explicit modeling of uncertainties and can be computationally intensive, especially when handling complex data distributions or large datasets. On the other hand, BNNs naturally handle uncertainty through their probabilistic weight sampling mechanism.

**Figure 1:**
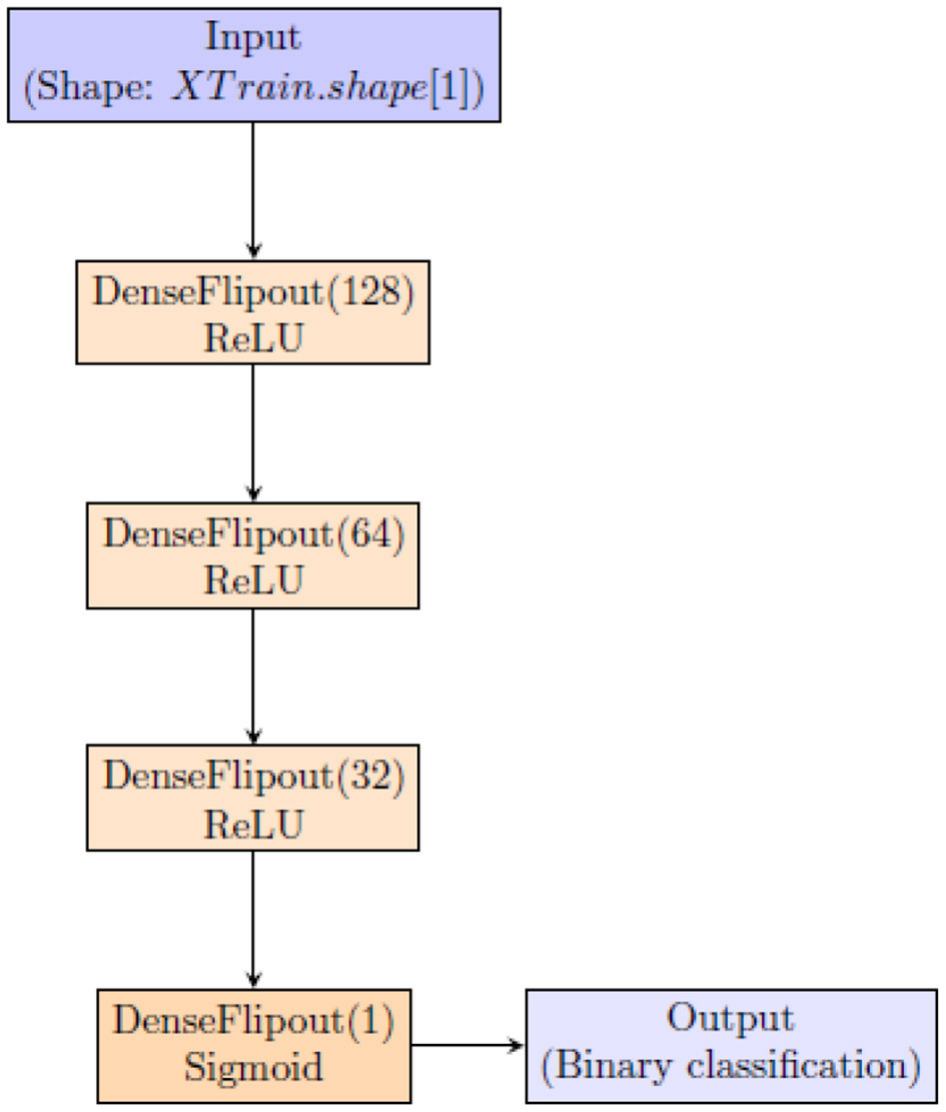
Architecture of the Bayesian Neural Network (BNN).

To quantify uncertainty using BNN, we employ the architecture shown in Figure 1.

This architecture uses tfpl.DenseFlipout layers (https://www.tensorflow.org/probability/api_docs/python/tfp/layers/DenseFlipout), which are components of TensorFlow Probability (TFP) designed to integrate uncertainty into neural network predictions. These layers employ Bayesian inference by introducing stochastic weights during training, enabling the model to quantify uncertainty in its predictions. This Bayesian approach is crucial for enhancing the reliability of the neural network's outputs, particularly in applications where understanding the confidence of predictions is essential. Incorporating tfpl.DenseFlipout layers allows the model to effectively account for uncertainty, resulting in more reliable and insightful predictions (Dillon et al., 2017; Abdar et al., 2021a).

#### 3.3.2 Deep neural networks

**Figure 2:**
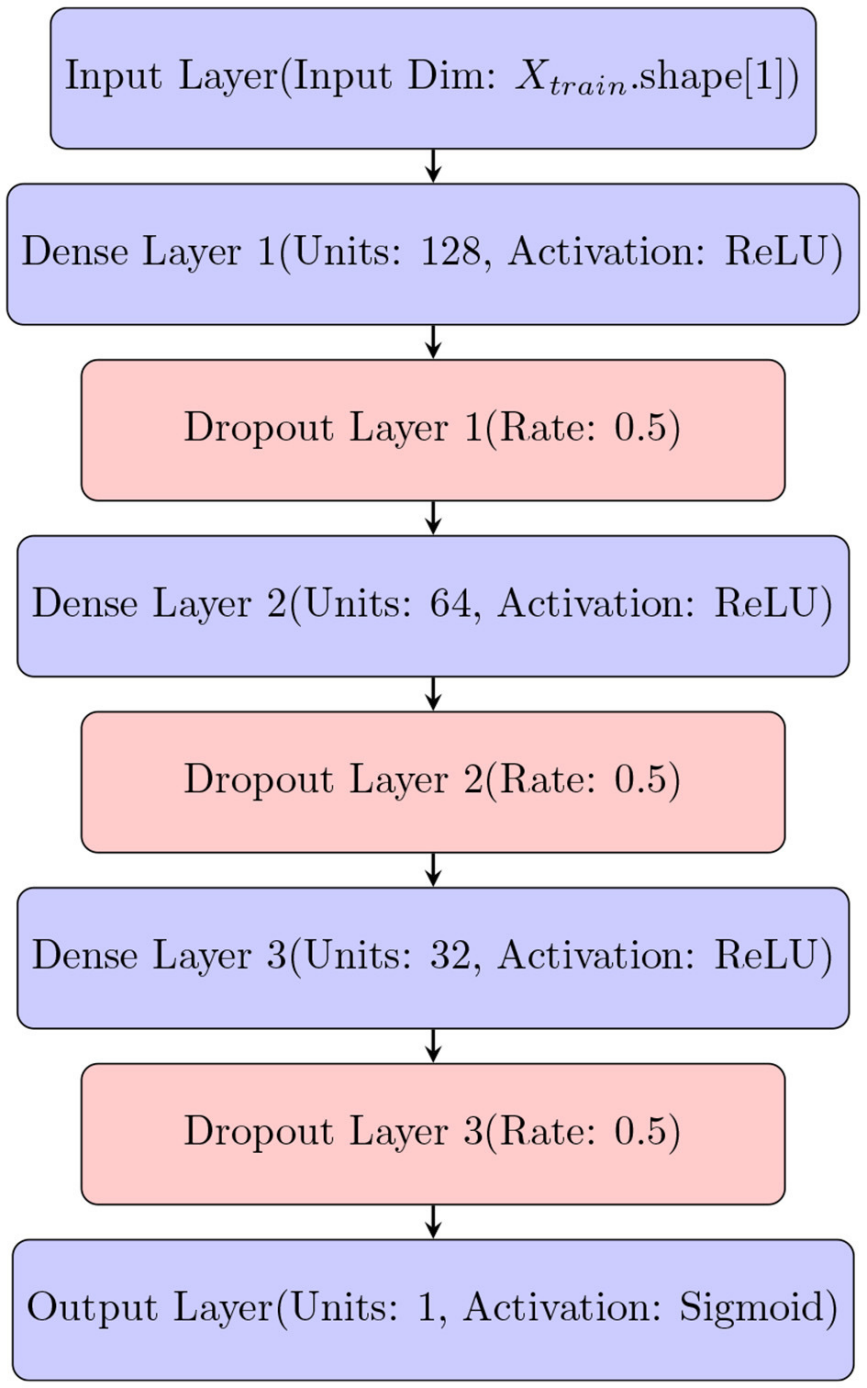
Architecture of Deep Neural Network (DNN) that is modified to quantify uncertainty using different dropout techniques.

To quantify uncertainty using DNN, we employ a practical approach to quantify uncertainty by leveraging various dropout techniques. We adapt a deep neural network (DNN) (Figure 2) to accommodate dropout techniques such as Monte-Carlo dropout.

Dropout techniques provide a means for estimating uncertainty in model predictions (Gal and Ghahramani, 2016; Kendall and Gal, 2017). Specifically, during inference or testing, dropout is applied stochastically to the network, resulting in multiple predictions for the same input. By averaging these predictions, we can obtain an estimate of the model's uncertainty (Srivastava et al., 2014; Gal and Ghahramani, 2016).

We explore different dropout techniques, such as Monte Carlo dropout, Ensemble dropout, and Expected Model Change dropout applied to DNNs, to comprehensively assess and quantify uncertainty in our multi-class classification task. In addition, we compare the performance of the modified DNN with different dropout techniques to that of the BNN.

### 3.4 Uncertainty quantification using Monte-Carlo (MC) dropout

We use the MC dropout technique as a regularization method for computing predictions during both the training and inference phases. By averaging multiple predictions, we aim to improve accuracy. As discussed earlier, estimating the posterior distribution poses computational challenges. To address this issue, we leverage MC sampling methods (Asgharnezhad et al., 2022). These methods involve performing multiple stochastic forward passes with dropout during testing, which generates MC samples from the posterior distribution. This approach reduces the computational burden of approximating the output posterior distribution.

In practice, the model's predictive mean resembles the expectation of ŷ (the predicted output). As a result, the final prediction for a test sample is obtained by using the predictive mean, denoted as μ_pred_, computed over the MC iterations (Ghoshal and Tucker, 2020).


(4) $$\begin{array}{l} \mu_{pred}=\frac{1}{T}\overset{T}{\underset{t=1}{\sum }}P\left(y=k|x,\omega \right) \end{array}$$

where the test input is denoted by *x*. The prediction probability produced from the softmax output is denoted by *P*(*y* = *k* ∣ *x*, ω). Additionally, ω denotes the model's parameters for the *i*th forward pass and the Monte Carlo (MC) iterations or forward passes are represented by *T*.

The output prediction for each test sample $\hat{x}$ is determined by selecting the class with the highest predictive mean, while the variance provides a measure of predictive uncertainty. Validating the model under epistemic uncertainty has traditionally been a complex task. In order to quantify epistemic uncertainty, Ghoshal and Tucker (2020) suggests utilizing predictive entropy (PE):


(5) $$\begin{array}{l} PE=-\underset{c}{\sum }P\left(y=k|x,\omega \right)\;logP\left(y=k|x,\omega \right) \end{array}$$

where *c* is the number of classes. Equation 5 provides a measure of the confidence of model in making predictions in classification tasks. Additionally, **PE** evaluates how much a prediction correlates to each individual class, or how much a prediction differs from its true label. A model is more confident in making predictions when the value of *PE* gets smaller.

### 3.5 Uncertainty quantification using Ensemble Monte-Carlo (EMC) dropout

Uncertainty quantification using Ensemble Monte Carlo (EMC) dropout entails employing a technique that combines ensemble methods with Monte-Carlo dropout. It utilizes an ensemble of different DNN architectures. When using the Monte Carlo dropout algorithm, multiple stochastic forward passes are performed to evaluate each network in the ensemble. The resulting posterior probabilities are averaged to estimate a single Gaussian distribution. The calculation of the predictive entropy (PE) metric is identical to that of the ensemble approach, with the only significant difference being the methodology that is used in determining the following posterior distribution;


(6) $$\begin{array}{l} \hat{P}\left(y|x\right)=\frac{1}{T}\overset{T}{\underset{t=1}{\sum }}\hat{P}\left(ŷ|\hat{x},\hat{\omega }\right) \end{array}$$

and the predictive entropy (PE) metric is then expressed as follows


(7) $$\begin{array}{l} PE=-\underset{c}{\sum }\hat{P}\left(y|x\right)\;log\hat{P}\left(y|x\right) \end{array}$$

where *C* is the number of classes and ω are the model's parameters.

### 3.6 Uncertainty quantification using Ensemble Bayesian Neural Network (EBNN)

The Ensemble Bayesian Network (EBNN) is a collection of networks that collaborate to perform a particular task. Each network generates predictive probabilities, that are veraged to obtain the final predictive probability. The predictive entropy (PE) (Equation 9) is again used to quantify the uncertainty.


(8) $$\begin{array}{l} \mu_{pred}=\frac{1}{T}\overset{T}{\underset{t=1}{\sum }}P_{\theta_{i}}\left(y=k|x,\omega \right) \end{array}$$

(9) $$\begin{array}{l} PE=-\underset{c}{\sum }P_{\theta_{i}}\left(y=k|x,\omega \right)\;logP_{\theta_{i}}\left(y=k|x,\omega \right) \end{array}$$

where *θ*_*i*_ is set of the *i*th network element's parameters, and *C* represents the number of classes. A smaller value of *PE* indicates similarity of the predictions from all individual networks.

### 3.7 Model training

We begin by extracting essential features from the CXR images by pretraining a DenseNet121 model using normal chest X-ray images and subsequently use the extracted features to evaluate whether DNNs have the potential to classify chest X-ray images. This approach, adapted from Asgharnezhad et al. (2022) is differnt from the conventional practice of training deep models from scratch in a transfer learning scenario. Instead, the approach fine-tunes the weights of pre-existing deep neural networks that are pretrained on natural image datasets such as ImageNet, which are specifically tailored for medical image analysis. Next, we utilize the methodology suggested by Alarab and Prakoonwit (2023) to perform One vs. All (OvA) classification of chest X-ray images of pneumonia, COVID-19 and normal images. Here, we transform the multi-class problem classification task into multiple binary classification problems by assigning temporary labels to the dataset, distinguishing each class from the rest of the data. This step involves training multiple two-class classifiers, each focused on discerning one class from the remaining *C*−1 classes. Thereafter, the Deep Neural Network (DNN) model is incorporated into our experimental procedure.

To achieve optimal performance during training, it is important to employ optimal hyper-parameters of the deep learning algorithms, particularly the learning rate (lr). According to Zhang et al. (2020), a lower lr enhances the reliability of the training phase but can prolong the optimization process due to smaller updates in the loss function. Conversely, a higher lr risks non-convergence or divergence, as it may cause the optimization phase to skip over the optimal value, worsening the loss function. This can lead to unproductive oscillations and poor generalization, as the training weights fail to stabilize at an optimal value. Following the recommendations in Kingma and Ba (2014), Zhang et al. (2020), Asgharnezhad et al. (2022), and Sun et al. (2024), who obtained the best values of all evaluation metric when we set the learning rate is set to 0.001, we used a default learning rate of 0.001 for the Adam algorithm, which is deemed effective for stochastic optimization.

The architecture of the deep neural network (DNN) used in this study is defined by the create_model() function, which generates the DNN model using TensorFlow (Abadi et al., 2015). The model is designed to include three hidden layers with 128, 64, and 32 neurons, respectively, and utilizes *ReLU* activation functions for non-linearity. Dropout layers with a dropout rate of 0.5 are included after each hidden layer to prevent overfitting. The output layer produces probabilistic predictions using a sigmoid activation function. The model is compiled with a binary cross-entropy loss function and optimized using the Adam optimizer (Kingma and Ba, 2014) with a learning rate of 0.001. This architecture is tailored for binary classification tasks and enables robust model performance through dropout-based regularization.

Table 1 presents the architecture of the DNN and the corresponding number of parameters.

**Table 1:** Architecture of the Deep Neural Network (DNN) and number of parameters.

**Layer**	**Number of parameters**
Input layer (Dense)	19,328
Dropout layer 1	0
Hidden layer 1	8,256
Dropout layer 2	0
Hidden layer 2	2,080
Dropout layer 3	0
Output layer (dense)	33
Total	29, 697

The DNN architecture iterates over each class label in the dataset and trains separate models for each binary classification task (e.g., Class 0 vs. All, Class 1 vs. All, etc.). For each class, the DNN model is trained using the training data and evaluated using the test data. The evaluation metrics include accuracy, F1 score, precision, recall, and ROC AUC score, which are computed and stored. This approach ensures a comprehensive assessment of the model's performance across different classification tasks.

The training parameters for the DNN are detailed in Table 2. The model was trained over 15 epochs with a batch size of 64. In addition, five different models were trained for Ensemble Monte Carlo (EMC) and Ensemble Bayesian neural network (EBNN) to enhance model robustness.

**Table 2:** Training parameters for DNN.

**Parameter**	**Value**
Learning rate	0.001
Epochs	15
Batch size	64
Number of models	5

After training and during the inference stage of DNN, we use different uncertainty quantification techniques to assess model performance independently. Monte Carlo Dropout (MC Dropout) involves maintaining dropout layers active during inference, enabling stochastic sampling of predictions by randomly deactivating units and their connections in each forward pass. This allows the model to generate multiple predictions per input, thereby, capturing the variance in the outcomes to estimate uncertainty. On the other hand, Ensemble Bayesian Neural Network (EBNN) averages predictions across multiple stochastic passes, thereby smoothing out prediction variability and providing a more stable estimate of uncertainty. In addition, Ensemble Monte Carlo Dropout (EMC) refines uncertainty estimation by aggregating predictions from an ensemble of models, each trained with dropout, to produce a consensus view of prediction uncertainty. These techniques individually yield probabilistic distributions that quantify uncertainty, offering more insights into the confidence level of the model's outputs. Such detailed uncertainty quantification is crucial in applications like medical diagnostics, where understanding prediction confidence supports informed decision-making.

Model training was conducted using Python libraries such as NumPy, PyTorch, and pandas. Each experiment was repeated 100 times with different random seeds to ensure repeatability. Table 3 shows the training times for DNN with different uncertainty quantification techniques, trained for 15 epochs with a batch size of 64.

**Table 3:** The table also shows the training times for DNN trained using Monte Carlo (MC) dropout, as well as the times for DNN trained with five different models of Ensemble Monte Carlo (EMC) and Ensemble Bayesian Neural Network (EBNN) dropout techniques.

**Model**	**Trainable parameters**	**Number of epochs**	**CPU elapsed time (min)**
DNN + MC dropout	29,697	15	13.618
DNN + EMC dropout	148,485	15	20.477
DNN + EBNN dropout	148,485	15	20.477

The experiments were executed on Google Colab, utilizing its default hardware settings, which include a GPU (1xTesla K80, compute 3.7, 2496 CUDA cores, 12GB GDDR5 VRAM).

The performance of DNN (with diferrent dropout techniques) was compared using model evaluation metrics described in Section 3.8.

### 3.8 Model evaluation metrics

#### 3.8.1 Traditional evaluation metrics

To assess the performance of DNN without the dropout techniques, we adopted several traditional evaluation metrics namely; sensitivity, specificity, accuracy, that have been used in previous biomedical papers (Hajian-Tilaki, 2013; Boughorbel et al., 2017; He et al., 2021; Helaly et al., 2022; Le and Xu, 2023; Nguyen Quoc Khanh Le, 2023). These metrics are defined by the following equations. We used the number of true positives (TP), true negatives (TN), false positives (FP), and false negatives (FN) to compute these metrics.


(10) $$\begin{array}{c} \text{Sensitivity}=\frac{TP}{TP+FN} \end{array}$$

(11) $$\begin{array}{c} \text{Specificity}=\frac{TN}{TN+FP} \end{array}$$

(12) $$\begin{array}{c} \text{Accuracy}=\frac{TP+TN}{TP+TN+FP+FN} \end{array}$$

(13) $$\begin{array}{c} \text{Precision}=\frac{TP}{TP+FP} \end{array}$$

where TP represents the true positives, TN represents the true negatives, and FP and FN represent the false positives and false negatives, respectively. Boughorbel et al. (2017) states that the MCC lies is in the interval [−1, 1], with 1 indicating perfect classification and -1 indicating perfect misclassification.

#### 3.8.2 Performance metrics for the predictive uncertainty estimations

The dropout techniques, including MC dropout, EMC, and EBNN, are incorporated into deep neural network (DNN) for uncertainty quantification. Subsequently, we utilize the uncertainty confusion matrix developed by Asgharnezhad et al. (2022), which employs a concept akin to a confusion matrix, to carry out the predictive uncertainty evaluation. In this study, we employ the “One 2 vs. All” multiclassification strategy, which reduces to binary classification for each class. The performance indicators for predictive uncertainty estimations are quantified by the uncertainty confusion matrix as shown in Table 4.

**Table 4:** Confusion matrix for calculating uncertainty quantification metrics (Asgharnezhad et al., 2022).

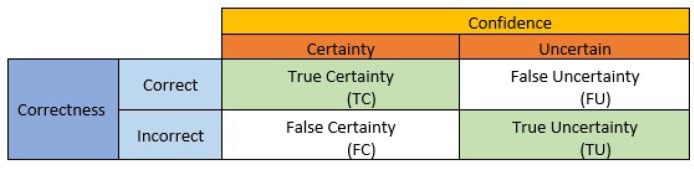

The uncertainty confusion matrix objectively and quantitatively evaluates the predictive uncertainty estimates. As shown in Table 4, the predictions are categorized into two groups, *correct* and *incorrect*, after being compared to the labels of the ground truth. Additionally, a threshold is employed to evaluate and categorize prediction uncertainty estimates into two groups: *uncertain* and *certain*.

Four different outcomes can result from the combination of correctness and confidence, as shown in Table 4 namely: (i) *true certainty (TC)*, which represents a combination of correct and certain predictions; (ii) *true uncertainty (TU)*, which represents a combination of incorrect and uncertain predictions, (iii) *False certainty (FC)* denotes forecasts that are certain but erroneous and, (iv) *false uncertainty (FU)* denotes predictions that are definite but incorrect. The intended results are the diagonal entries *TC* and *TU*. These outcomes are referred to as True Negative (TN) and True Positive (TP) in the standard confusion matrix, respectively. The following quantitative performance metrics that purely and objectively quantify the prediction uncertainty estimations are produced as a result of these combinations of correctness and confidence groups:

(i) Uncertainty sensitivity *(USen*):
(14) $$\begin{array}{c} USen=\frac{TU}{TU+FC} \end{array}$$
  The classic confusion matrix's sensitivity (*recall*) or true positive (TP) rate corresponds to *USen* or (*URec*), respectively. *USen* is very important in that it quantifies the model's power to express confidence in incorrectly classified samples.
(ii) Uncertainty Specificity (*USpe*):
(15) $$\begin{array}{c} USpe=\frac{TC}{TC+FU} \end{array}$$
  USpe is equivalent to the specificity performance metric derived from the tradional confusion matrix.
(iii) Uncertainty precision (*UPre*):
(16) $$\begin{array}{c} UPre=\frac{TU}{TU+FU} \end{array}$$
  UPre is equivalent to precision derived from the traditional confusion maytrix
(iv) Uncertainty accuracy (UAcc):
(17) $$\begin{array}{c} UAcc=\frac{TU+TC}{TU+TC+FU+FC} \end{array}$$
  A model with a high *UAcc* is considered reliable.

Other metrics that are used to evaluate the performance of the models include the area under curve receiver operating curve (AUC-ROC), confidence, and the expected calibration error (ECE) described below.

(v) AUC-ROC:
  The results of the classification task are further cross-validate using the area under curve receiver operating curve (AUC-ROC). AUC-ROC is a probability curve that represents a degree or measure of separability. This means that we can measure how a model can distinguish between classes. The AUC-ROC is a function of sensitivity and specificity.
(vi) Expected Calibration Error (ECE) and Brier Score:
  Predictions are categorized into different *M bins* (based on the value of the maximum softmax output) according to their confidence in order to calculate the ECE. The calibration errors in each bin quantify the discrepancy between the percentage of correctly classified predictions (accuracy) and the probability average (confidence). The calisensitivity (*U*Sensebration errors across all bins are weighted to produce the ECE.
(18) $$\begin{array}{c} ECE=\overset{M}{\underset{m=1}{\sum }}\frac{\left|B_{m}\right|}{n}|acc\left(B_{m}\right)-conf\left(B_{m}\right)| \end{array}$$
  where *acc*(*B*_*m*_) and *conf*(*B*_*m*_) are the accuracy and confidence in the *m-th* bin:

Additionally, we examine the Brier score, as described in Brier (1950). The Brier score is a metric used to evaluate the accuracy of probabilistic predictions made by a model. A lower Brier score indicates better calibration and accuracy of the model's predictions.

## 4 Results and discussions

### 4.1 DNN without uncertainty quantification

Features were extracted from chest X-ray (CXR) images in the Pneumonia, Normal, and COVID-19 datasets using two distinct pre-trained DenseNet models. For the Pneumonia and Normal datasets, we employed the DenseNet121 model, trained on various sources, including the RSNA Pneumonia Challenge, CheXpert, and normal CXR images. Each CXR image was resized to a standard dimension of 224 × 224 pixels, converted into an array, and pre-processed to meet the model's input requirements. The images were then processed through the DenseNet121 model, from which features were extracted from the feature layer and subsequently flattened into a one-dimensional vector.

For the COVID-19 dataset, we utilized a DenseNet model with “all” weights. The images were read in grayscale, resized to 224 × 224 pixels, and passed through the PyTorch-implemented DenseNet model. Features were extracted from the feature layer, detached from the computational graph, converted to a NumPy array, and flattened into a one-dimensional vector. These feature vectors, along with their corresponding labels (Normal: “Label”: Class 0, COVID-19: “Label”: Class 1, Pneumonia: “Label”: Class 2) and filenames, were systematically organized in a Pandas DataFrame (Features), which was subsequently used for model training and UQ quantification using different techniques.

After pretraining a DenseNet121 model using normal chest X-ray images to extract essential features, we trained DNN on Class 0 vs. All, on Class 1 vs. All, and on Class 2 vs. All without uncertainty quantification to give a normal confusion matrix and subsequently evaluate the performance metrics for each class.

Table 5 shows the results of the one vs. all classifications without uncertainty quantification.

**Table 5:** Results of the “One vs. All” (OvA) multi-class classification of chest-xray images using DNN without uncertainty quantification.

**Class**	**AUC-ROC**	**Accuracy (%)**	**F1 score (%)**	**Sensitivity (%)**	**Specificity (%)**	**Precision (%)**
Class 0 vs. all	96.5	92.8	90.0	90.4	94.2	89.7
Class 1 vs. all	97.3	93.4	90.6	91.7	94.3	89.6
Class 2 vs. all	93.6	91.7	85.4	83.5	95.1	89.9

Table 5 shows that DNN has high discriminative performance across all classes, with AUC-ROC values of 96.5% for normal images, 97.3% for COVID-19 images, and 93.6% for pneumonia images. The model's high AUC-ROC values indicate excellent effectiveness in discriminating each class from others. The model achieves good overall accuracy, with 92.8% for normal images, 93.4% for COVID-19 images, and 91.7% for pneumonia images, demonstrating effective classification across image types. Sensitivity and specificity measures are important in real-world applications, especially in the medical industry. The DNN accurately detected normal images with a sensitivity of 90.4% and specificity of 94.2%, minimizing false positives. For COVID-19 images, the sensitivity is 91.7% and specificity is 94.3%, underscoring the model's ability to accurately detect COVID-19 cases while minimizing the misclassification of other conditions as COVID-19. In the case of pneumonia images, the sensitivity is slightly lower at 83.5%, but the specificity remains high at 95.1%, demonstrating the model's effectiveness in identifying true pneumonia cases and reducing the likelihood of misclassifying other conditions as pneumonia.

These results have significant implications for real-life healthcare settings. High sensitivity in detecting COVID-19 and pneumonia images is crucial for early diagnosis and treatment, which can prevent the spread of infections and mitigate complications. On the other hand, high specificity across all classes ensures that healthy individuals are not subjected to unnecessary medical interventions, while patients receive appropriate treatments for their conditions. Additionally, high precision for each class indicates that the model reliably identifies true cases, enhancing trust in the diagnostic process and ensuring efficient allocation of medical resources. Overall, the DNN's strong performance metrics suggest its potential as a valuable tool in medical diagnostics, contributing to improved patient care and public health outcomes.

While the results demonstrate that DNN is suitable for “OvA” multiclassifying tasks, it lacks the ability to quantify uncertainty, which can be crucial in certain scenarios such as identifying for example chest X-rays of pneumonia patients. To quantify predictive uncertainty, we start by assessing the calibrations of the predictions produced by the DNN.

### 4.2 Expected calibration error

The expected calibration error (ECE) values for the different multi-class classification tasks are presented in Table 6. These ECE values, derived from 19, provide a quantitative evaluation of the alignment between the actual outcomes and the predicted probabilities by the DNN. For Class 0 vs. All, Class 1 vs. All, Class 2 vs. All the ECE values are 0.0134, 0.0128, and 0.0130, respectively. These ECE values are much lower, indicating better calibration by the DNN. This shows that the probabilities that are predicted by the DNN are closely aligned with the actual outcomes across the three multi-class classification scenarios.

**Table 6:** Calibration metrics for each class.

**Class**	**ECE**	**Brier score**
Class 0 vs. All	0.0134	0.123
Class 1 vs. All	0.0128	0.234
Class 2 vs. All	0.0130	0.345

In addition, Table 6 presents the Brier scores that evaluate the DNN's calibration and accuracy performances across the multi-class classification scenarios. For Class 0 vs. All, the Brier score is 0.123, indicating that predictions are well-calibrated and accurate. For Class 1 vs. All, the Brier score of 0.234 is slightly higher, showing that the calibration and accuracy are reasonable, with some indication of uncertainty. On the other hand, Class 2 vs. All has the highest Brier score (0.345), suggesting that it may be more challenging to predict this class correctly than the others.

To quantify uncertainty, we employ a specialized type of DNN called the Bayesian neural network (BNN). This BNN incorporates various dropout techniques, including Monte Carlo (MC) dropout, Ensemble Monte Carlo (EMC), and Ensemble Bayesian neural networks (EBNN) to quantify uncertainty.

### 4.3 Uncertainty quantification using Bayesian neural networks

The results presented in Table 7 reveal varying performance metrics across different classes in the “One vs. All” (OvA) multi-class classification of chest X-ray images. For Class 0 vs. All, the model achieves an AUC-ROC of 89.8%, indicating strong discriminative ability in distinguishing normal images from others, supported by high sensitivity (82.2%) and specificity (83.0%). However, the F1 score of 77.4% suggests a moderate balance between precision and recall. In contrast, Class 1 vs. All shows a lower AUC-ROC of 79.2%, reflecting greater difficulty in accurately identifying COVID-19 cases, with sensitivity and precision at 77.1 and 72.1%, respectively. Class 2 vs. All exhibits challenges in achieving precision (F1 score 58.9%), despite a reasonable AUC-ROC of 77.6% and high specificity (84.3%).

**Table 7:** Comparison of the uncertainty-aware evaluation metrics produced by DNN with uncertainty quantification techniques (MC Dropout, EBNN, Ensemble, and EMC Dropout) for Class 0 vs. All.

**Model**	***U*Acc**	***U*F1 Score**	***U*Prec**	***U*Sens**	***U*Spec**	***U*AUC-ROC**	**Brier score**
MC Dropout	81.0	75.5	70.3	81.5	80.7	87.9	0.168
EBNN	92.6	90.0	86.9	93.3	92.1	95.0	0.157
EMC Dropout	87.5	80.7	90.7	72.6	95.9	95.1	0.178

### 4.4 Uncertainty quantification using deep neural networks

Uncertainty quantification was performed for each of Class 0 vs. All, Class 1 vs. All, and Class 2 vs. All. Table 7 presents a comparison of the uncertainty-aware evaluation metrics produced by the BBNN, MC Dropout, EBNN, Ensemble, and EMC Dropout uncertainty quantification techniques for Class 0 vs. All.

#### 4.4.1 Class 0 vs. all

The results show that EBNN outperforms the other models achieving the highest *U*Acc of 92.6% and a *U*AUC-ROC value of 87.9%, indicating its superior ability in accurately classifying the OvA instances while quantifying uncertainty. EBNN also demonstrates superiority across other metrics such as Brier Score, with a score of 0.1567, re-affirming its overall effectiveness in predictive uncertainty quantification.

*U*F1 Score, *U*Prec, *U*Sens, and *U*Spec were also employed to quantify prediction uncertainty. These metrics are important because they provide insights into a model's ability to make predictions. In this study we used a threshold of 0.30, following Asgharnezhad et al. (2022)'s recommendation, to calculate these performance metrics. The best-performing model, EBNN, showed outstanding performance across these metrics: *U*F1 Score = 90.0%, *U*Prec = 86.9%, *U*Sens = 93.3%, and *U*Spec = 92.1%. These results emphasize EBNN's reliability and accuracy in estimating uncertainty.

The results in Table 8 show that the Bayesian Neural Network (BNN) employed for “One vs. All” multi-class classification of chest X-ray images exhibits varying performance across classes, with the highest accuracy (82.7%) achieved by Class 0 vs. All. In addition, Class 0 vs. All's AUC-ROC is 89.8%, indicating better discriminative ability compared to the other classes. However, Class 2 vs. All achieved the lowest F1 score of 58.9%, suggesting it had challenges in correctly identifying the true positives. The Brier Scores ranged from 0.161 to 0.194, reflecting reasonable but not perfect calibration of predicted probabilities across all classes.

**Table 8:** Results of the “One vs. All” (OvA) multi-class classification of chest-X ray images using Bayesian Neural Networks (BNN).

**Class**	***U*Acc**	***U*F1 Score**	***U*Prec**	***U*Sens**	***U*Spec**	***U*AUC-ROC**	**Brier score**
Class 0 vs. All	82.7	77.4	73.0	82.2	83.0	89.8	0.161
Class 1 vs. All	72.9	69.0	72.1	77.1	92.2	79.2	0.194
Class 2 vs. All	76.6	58.9	60.0	57.8	84.3	77.6	0.194

Notably, Table 9, shows that the different multi-class classification models perform differently quantifying the percentage uncertainty in chest X-ray image classification for the Class 0 vs. All case.

**Table 9:** Comparison of the percentage uncertainty (%) in the different uncertainty-aware evaluation metrics produced by the DNN, MC Dropout, EBNN, Ensemble, and EMC Dropout uncertainty quantification techniques for Class 0 vs. All.

**Model**	***U*Acc (%)**	***U*ROC-AUC**	**UPrec (%)**	**USens (%)**
MC Dropout	11.8	8.6	19.4	8.9
EBNN	0.2	1.5	2.8	2.9
EMC Dropout	5.3	1.4	1.0	17.8

EBNN emerges as the best-performing model, achieving the lowest uncertainty values across several performance metrics for Class 0 vs. All. Specifically, EBNN achieves the lowest uncertainty in accuracy (*U*Acc = 0.2%), signifying its robustness in making accurate predictions. Moreover, for Class 0 vs. All, EBNN exhibits high discriminatory power with the lowest uncertainty in *U*ROC-AUC = 1.5%. Furthermore, EBNN produces outstanding performance not only in achieving lower uncertainty in overall accuracy but also in achieving the lowest percent uncertainty in precision (*U*Prec = 2.8%). This indicates that the model correctly identifies Class 0 instances among all positive predictions. Additionally, EBNN produces the lowest percent uncertainty in sensitivity (*U*Sens = 2.9%), demonstrating its effectiveness in capturing the true positive instances of Class 0 while reducing false negatives.

The superior performance of EBNN's indicates that it provides more reliable uncertainty estimates across various key metrics compared to other models. This enhances its suitability for deployment in critical healthcare applications.

#### 4.4.2 Class 1 vs. all

Table 10 presents a comparison of the uncertainty-aware evaluation metrics produced by the DNN, MC Dropout, EBNN, Ensemble, and EMC Dropout uncertainty quantification techniques for the Class 1 vs. All scenario.

**Table 10:** Comparison of the uncertainty-aware evaluation metrics produced by the DNN, MC Dropout, EBNN, Ensemble, and EMC Dropout uncertainty quantification techniques for Class 1 vs. All.

**Model**	***U*Acc**	***U*F1 Score**	***U*Prec**	***U*Sens**	***U*Spec**	***U*AUC-ROC**	**Brier score**
MC Dropout	74.7	53.1	76.0	59.9	93.0	80.0	0.189
EBNN	70.7	73.7	82.4	71.4	97.5	93.4	0.182
EMC Dropout	83.5	72.3	88.0	61.7	95.5	95.8	0.165

Table 10 shows that the EMC Dropout model is the best-performing model for Class 1 vs. All classification, as indicated by the different performance evaluation metrics. The model achieved a *U*Acc of 83.5% and a *U*F1 Score of 72.3%, indicating its accuracy in correctly classifying Class 1 vs. All instances. In addition, the model has superior *U*Prec (88.0%) and *U*Spec values (95.5%), showing good predictive performance. Although its *U*Sens is relatively lower at 61.7% compared to other metrics, it still demonstrates solid sensitivity. The high *U*AUC-ROC value of 95.8% and a low Brier Score of 0.165 further indicate that the EMC Dropout model performs well in predictive uncertainty estimation.

Table 11 summarizes the percentage uncertainty for different metrics produced by the uncertainty quantification techniques for the Class 1 vs. All scenario.

**Table 11:** Comparison of the percent uncertainty (%) in the different uncertainty-aware evaluation metrics produced by the DNN, MC Dropout, EBNN, Ensemble, and EMC Dropout uncertainty quantification techniques for Class 1 vs. All.

**Model**	***U*Acc (%)**	***U*ROC-AUC**	**UPrec (%)**	**USens (%)**
MC Dropout	18.7	17.3	13.6	31.8
EBNN	22.7	3.9	7.2	20.3
EMC Dropout	9.9	1.5	1.6	30

The results show that EMC Dropout emerges as the best-performing model by achieving the lowest uncertainty percentage values across most metrics for Class 1 vs. All. Specifically, EMC Dropout achieves the lowest uncertainty in accuracy (*U*Acc = 9.9%), *U*ROC-AUC (1.5%), precision (*U*Prec = 1.6%), and sensitivity (*U*Sens = 30%). The results indicate that the EMC Dropout model is capable of providing more reliable uncertainty estimates compared to other models.

#### 4.4.3 Class 2 vs. all

Table 12 presents a comparison of the uncertainty-aware evaluation metrics produced by the DNN, MC Dropout, EBNN, Ensemble, and EMC Dropout uncertainty quantification techniques for Class 2 vs. All. The Ensemble Bayesian Neural Network (EBNN) model outperforms the other models evaluated for Class 2 vs. All classification, showing very good classification performance across various evaluation metrics. EBNN accurately classifies Class 2 and Rest instances, with an *U*Accuracy of 87.8% and a *U*F1 Score of 75.5%. Also, it has a high *U*Prec 89.9%, which indicates accurate positive cases predictions, and a *U*Spec of 97.00%, which highlights its accuracy in identifying negative cases. Nevertheless, It is noteworthy that its *U*Sens, is just 65.1%, indicating room for improvement in classifying all positive instances. This results show that EBNN model performs very well in predictive uncertainty estimation, as evidenced by its high *U*AUC-ROC value of 0.91 and comparatively low Brier Score of 0.19. These results confirm the model's reliability and efficiency in handling uncertain predictions for Class 2 vs. All classification tasks. Futhermore, we quantified the percentage uncertainty produced by the different uncertainty quantification techniques across different evaluation metrics and the results are shown in Table 13.

**Table 12:** Comparison of the uncertainty-aware evaluation metrics produced by the DNN, MC Dropout, EBNN, Ensemble, and EMC Dropout uncertainty quantification techniques for Class 2 vs. All.

**Model**	***U*Acc**	***U*F1 Score**	***U*Prec**	***U*Sens**	***U*Spec**	***U*AUC-ROC**	**Brier Score**
MC Dropout	74.4	53.9	56.5	51.6	83.7	75.8	0.196
EBNN	87.8	75.5	87.5	65.1	97.0	90.6	0.185
EMC Dropout	71.8	65.0	77.2	62.8	91.0	84.4	0.190

**Table 13:** Comparison of the percentage uncertainty (%) in the different uncertainty-aware evaluation metrics produced by the DNN, MC Dropout, EBNN, Ensemble, and EMC Dropout uncertainty quantification techniques for Class 2 vs. All.

**Model**	***U*Acc (%)**	***U*ROC-AUC**	**UPrec (%)**	**USens (%)**
MC Dropout	17.3	17.8	31.0	31.9
EBNN	3.9	3.0	2.4	18.4
EMC Dropout	19.9	9.2	12.7	20.7

The results in Table 13 indicate that EBNN has the lowest percentage uncertainty across all the different evaluation metrics (*U*Acc =12.766%, UAUC-ROC = 9.405%, *U*Prec = 10.127%, and *U*Sens = 34.862%).

## 5 Discussion

This paper presented an in-depth quantification of uncertainty across different evaluation metrics using various uncertainty quantification methods deployed on multi-class classification tasks: Class 0 vs. All, Class 1 vs. All, and Class 2 vs. All. A chest X-ray image dataset with three classes (COVID-19, Pneumonia, and Normal images) was used to perform the multi-class classification tasks. Several uncertainty quantification techniques (MC dropout, EBNN, Ensemble, and EMC dropout) were employed for all classifications in chest X-ray analysis. Through the evaluation of these techniques, the percentage uncertainty estimates were obtained for each method across different evaluation metrics. The quantified uncertainty provides insights into the reliability and predictive performance of these quantification techniques.

A comparative analysis of Bayesian Neural Networks (BNN) and Deep Neural Networks with Uncertainty Quantification (DNN with UQ) techniques for multi-class classification of chest X-ray images shows notable differences in performance metrics for Class 0 vs. All, Class 1 vs. All, and Class 2 vs. All scenarios. For Class 0 vs. All, the EBNN method achieved a *U*Acc of 92.6%, *U*AUC-ROC of 95.0%, and a Brier Score of 0.157, significantly outperforming the BNN's Acc of 82.7%, AUC-ROC of 89.8%, and Brier Score of 0.161. Similarly, for Class 1 vs. All, the EMC Dropout technique showed superior results with a *U*Acc of 83.5%, *U*AUC-ROC of 95.8%, and a Brier Score of 0.165, compared to the BNN's Acc of 72.9%, AUC-ROC of 79.2%, and Brier Score of 0.194. In the Class 2 vs. All scenario, the EBNN also excelled with a *U*Acc of 87.8%, *U*AUC-ROC of 90.6%, and a Brier Score of 0.185, versus the BNN's Acc of 76.6%, AUC-ROC of 77.6%, and Brier Score of 0.194.

Across all classes, DNNs with UQ techniques, especially EBNN and EMC Dropout, consistently demonstrated superior performance metrics compared to BNNs. They achieved higher *U*Acc and *U*AUC-ROC values, indicating better classification accuracy and discriminative capability. Additionally, these models reported lower Brier Scores, reflecting more accurate probabilistic predictions. Further analysis of metrics such as F1 Score, Precision, Sensitivity, and Specificity revealed that DNNs with UQ maintained a better balance between precision and recall and exhibited greater robustness in identifying both positive and negative cases. Overall, the advanced DNNs with UQ not only provided enhanced performance but also ensured reliable uncertainty quantification, making them more suitable for critical healthcare applications like chest X-ray image classification.

## 6 Conclusion

This study made important contributions to the existing literature by providing novel insights into uncertainty quantification in the classification of COVID-19, particularly by extending the binary classification of COVID-19 task to the multi-class classification task. In addition, we have demonstrated that uncertainty-aware estimates of evaluation metrics can effectively be obtained from uncertainty quantification techniques across different multi-class classification scenarios, particularly in the context of medical image analysis. For example, the EBNN demonstrated superior performance in quantifying uncertainty, paving the way for improved model reliability and interpretability.

This study has offered substantial clinical relevance by integrating advanced UQ techniques into deep neural networks (DNNs), thereby significantly enhancing the interpretability and reliability of diagnostic predictions an essential factor for clinical decision-making. With UQ, clinicians can better gauge the confidence in model predictions, enabling more informed decisions regarding patient referrals and treatment plans. For instance, if a DNN model indicates high uncertainty in classifying a chest X-ray, it prompts clinicians to investigate further or refer the patient to a specialist, potentially leading to earlier diagnosis and intervention.

Accurately estimating the uncertainty associated with predictions helps mitigate the risks of misdiagnosis, especially in critical conditions like COVID-19 and pneumonia, where timely and accurate diagnosis is vital. Our findings demonstrate that models that are equipped with UQ achieve higher accuracy and thus, offer probabilistic predictions that may guide clinical actions more effectively than traditional models. Implementing the UQ techniques employed in this study in clinical settings can significantly improve diagnostic outcomes and patient care, underscoring its clinical implications and value in medical practice.

A limitation of this study is that uncertainty quantification relies on a specific chest X-ray image dataset, and the results may not generalize well to other image datasets. For future studies, we will explore the use of different image datasets, model architectures, and training strategies to investigate their impact on uncertainty quantification in the multi-class classification of COVID-19.

## Data Availability

Publicly available datasets were analyzed in this study. This data can be found at: https://www.kaggle.com/datasets/prashant268/chest-xray-covid19-pneumonia.
